# Combined intracavitary thermotherapy with iron oxide nanoparticles and radiotherapy as local treatment modality in recurrent glioblastoma patients

**DOI:** 10.1007/s11060-018-03005-x

**Published:** 2018-12-01

**Authors:** Oliver Grauer, Mohammed Jaber, Katharina Hess, Matthias Weckesser, Wolfram Schwindt, Stephan Maring, Johannes Wölfer, Walter Stummer

**Affiliations:** 1 Department of Neurology, University Hospital of Münster, Albert-Schweitzer-Campus 1, Building A1, 48149 Münster, Germany; 2 Department of Neurosurgery, University Hospital of Münster, Münster, Germany; 3 Institute of Neuropathology, University Hospital of Münster, Münster, Germany; 4 Department of Nuclear Medicine, University Hospital of Münster, Münster, Germany; 5 Institute of Radiology, University Hospital of Münster, Münster, Germany; 6 Department of Radiation Oncology, University Hospital of Münster, Münster, Germany; 7 Present Address: Competence Center for Neurosurgery, Hufeland Klinikum GmbH, Langensalzaer Landstraße 1, 99974 Mühlhausen, Germany

**Keywords:** Superparamagnetic iron oxide nanoparticles, Thermotherapy, HSP70, Caspase-3, PD-L1

## Abstract

**Background:**

There is an increasing interest in local tumor ablative treatment modalities that induce immunogenic cell death and the generation of antitumor immune responses.

**Methods:**

We report six recurrent glioblastoma patients who were treated with intracavitary thermotherapy after coating the resection cavity wall with superparamagnetic iron oxide nanoparticles (“NanoPaste” technique). Patients underwent six 1-h hyperthermia sessions in an alternating magnetic field and, if possible, received concurrent fractionated radiotherapy at a dose of 39.6 Gy.

**Results:**

There were no major side effects during active treatment. However, after 2–5 months, patients developed increasing clinical symptoms. CT scans showed tumor flare reactions with prominent edema around nanoparticle deposits. Patients were treated with dexamethasone and, if necessary, underwent re-surgery to remove nanoparticles. Histopathology revealed sustained necrosis directly adjacent to aggregated nanoparticles without evidence for tumor activity. Immunohistochemistry showed upregulation of Caspase-3 and heat shock protein 70, prominent infiltration of macrophages with ingested nanoparticles and CD3^+^ T-cells. Flow cytometric analysis of freshly prepared tumor cell suspensions revealed increased intracellular ratios of IFN-γ to IL-4 in CD4^+^ and CD8^+^ memory T cells, and activation of tumor-associated myeloid cells and microglia with upregulation of HLA-DR and PD-L1. Two patients had long-lasting treatment responses > 23 months without receiving any further therapy.

**Conclusion:**

Intracavitary thermotherapy combined with radiotherapy can induce a prominent inflammatory reaction around the resection cavity which might trigger potent antitumor immune responses possibly leading to long-term stabilization of recurrent GBM patients. These results warrant further investigations in a prospective phase-I trial.

## Introduction

For recurrent glioblastoma patients, there is increasing interest in local tumor ablative treatment modalities that can induce immunogenic cell death leading to the generation of tumor-specific immune responses.

Hyperthermia in tumor tissues induced by superparamagnetic iron oxide nanoparticles (SPIONs) subjected to an alternating magnetic field (AMF) has been evaluated by various ex-vivo and in-vivo experiments including assessments of biocompatibility, depot stability, and preclinical efficacy [1–7]. Moreover, in preclinical glioma models, local hyperthermia generated by SPIONs was shown to induce potent antitumor immune responses [8–10]. Immunohistochemical assays revealed enhanced infiltration of NK cells, macrophages, dendritic cells, and CD4^+^ and CD8^+^ T cells into the necrotic areas after thermotherapy. Interestingly, prominent leucocyte infiltrates could also be observed in unheated distant tumor portions similar to the abscopal effect described as a response to local radiotherapy [11].

Further investigation demonstrated that heat shock proteins (HSPs), including HSP70, trigger potent antitumor immunity during magnetic hyperthermia by the release of HSP-peptide complexes from dying tumor cells which were able to stimulate professional antigen-presenting cells (APCs) such as dendritic cells. This in turn leads to the induction of antigen-specific CD4^+^ and CD8^+^ T-cell responses [9, 12]. More recently, it could be shown that T-cell responses after hyperthermia are dominated by T-cells directed towards a limited number of epitopes, and that T-cells specific for these few epitopes frequently use a restricted T-cell receptor repertoire [13]. Collectively, these data indicate that intratumoral hyperthermia could be an ideal tool for the development of in-situ vaccination strategies in the treatment of glioblastoma patients.

Early clinical trials have demonstrated the efficacy and safety of intratumoral thermotherapy using SPIONs covered by an aminosilane type shell (NanoTherm®, MagForce AG, Berlin, Germany) combined with external beam radiotherapy in patients with recurrent glioblastoma [14, 15]. Histological analysis of tumor samples of treated patients showed that most of the injected nanoparticles were aggregated, and that the distribution of necrotic regions within the tumor were restricted to the sites of local instillation [16].

The previously advocated technique of stereotactic nanoparticle instillation suffers from considerable technical imponderabilities [14]. Therefore, we created a technique to “paste” or “plaster” the walls of the resection cavities with sufficiently high SPION concentrations for subsequent heating in an AMF (“NanoPaste” technique, see below). Using this technique, we treated six patients with recurrent glioblastoma. Here we report on tolerability and efficacy of the treatment and provide first insights into the mechanism of action of intracavitary thermotherapy.

## Materials and methods

### Patients

Six glioblastoma patients (n = 3 at first recurrence, n = 2 at second, and n = 1 at fourth recurrence) with a median age of 60 years (range 42–75 years) were treated with intracavitary thermotherapy. 2/6 tumors had a methylated MGMT promotor (33.3%). MGMT promoter status was determined in tissue obtained from primary operation. All candidates were primarily considered candidates for surgical resection and were offered intracavitary thermotherapy as an adjunctive treatment modality. Written informed consent was obtained from each patient. All patients consented to the scientific use of their biosamples. Analysis of biosamples was further approved by the ethical committee of the University of Muenster Medical School (2010-461-f-S).

### Nanoparticles

The magnetic fluid MFL AS-1 (NanoTherm®, MagForce AG, Berlin, Germany) consists of an aqueous dispersion of superparamagnetic nanoparticles with an iron concentration of 112 mg/ml. The nanoparticles are formed as magnetite (Fe_3_O_4_) cores of approx. 12 nm diameter coated with aminosilane, a bioinert, enzymatically not cleavable silicium compound with positively charged surface. It provokes fast adsorption to negatively loaded tissue proteins; the resulting deposits having been shown to remain stable for > 4 years (MagForce AG, undisclosed patient data). The fluid is manufactured according to European medical device regulations and has been approved in Europe for “use in brain tumors” since May 2010.

### Neurosurgical procedure and instillation of nanoparticles

Surgery was performed using standard techniques and 5-ALA-induced tumor fluorescence after administration of 20 mg/kg 5-ALA (Gliolan, Medac, Wedel, Germany). After tumor resection, the wall of the cavity was coated with two or three layers of NanoTherm® using a hydroxycellulose mesh and fibrin glue to increase the stability of the nanoparticle film and to create high local particle concentrations. Additionally, a closed-end thermometry catheter (MagForce AG, Berlin, Germany) was led through the particle deposits to allow for optoelectric temperature measurement.

### Treatment planning

A post-operative CT scan was fused with pre-operative MRI and post-operative 18F-FET-PET, the latter being performed to identify residual tumor tissue at the margins of the resection cavity [17]. Image fusion capability is included in the NanoPlan® treatment simulation software (MagForce AG, Berlin, Germany) and was performed using an algorithm based on the VTK/ITK framework [18]. The main determinants of tissue heat generation are nanoparticle density and tissue perfusion. Based on their specific absorption rate [19], particle distribution was determined by radiodensity measurement in the post-operative CT scan. Tissue perfusion had to be estimated, because to date there is no method available to reliably determine local tissue perfusion over the course of a hyperthermia application. NanoPlan® is able to simulate heat generation within the target volume as a function of AMF strength using the bioheat transfer equation, nanoparticle distribution as determined by semi-automatic CT image segmentation, and the assumption of tissue perfusion [18, 19]. As it is not yet technically possible to obtain a three-dimensional temperature chart during hyperthermia, the thermometry catheter, which is inserted through the whole target zone, provides at a least a one-dimensional excerpt from such a chart. The simulated temperature profiles along the catheter were continuously matched with the actual measurements during hyperthermia. Currently, the highest temperature along the catheter course, levelling out towards the rim of the target area, was used to fine-tune the strength of the AMF.

### Thermotherapy

Thermotherapy was performed in the alternating magnetic field applicator MFH-300F (NanoActivator®, MagForce AG, Berlin, Germany), operating at a frequency of 100 kHz and with variable magnetic field strengths of 2.5–15 kA/m. The applicator complies with the safety criteria for medical use imposed by the respective European authorities. Thermotherapy generally consisted of six semi-weekly sessions, and each thermotherapy session lasted 1 h. During the first session, the procedure was monitored using direct temperature measurements from the previously placed thermometry catheter. The first 1-h thermotherapy session was scheduled 3 days before the start of radiotherapy, while another five sessions were conducted at days 1, 4, 8, 11, 15 ± 1 day. Radiotherapy took place on the same day within a short time interval of 2 h as suggested by previous investigators [20].

### Radiotherapy

All patients had recurrent tumors and had been irradiated before with a median interval of 8.1 months (range 5.3–14.5 months) and standard doses of 60 Gy over the target volume. Four patients were eligible for fractionated intensity-modulated and image-guided radiotherapy. Radiotherapy was performed using a Varian TrueBeam™ linear accelerator or an Accuracy TomoTherapy HighArt HD II™. The planning target volume enclosed the resection cavity and FET-PET-positive tumor area with an additional margin of 3–5 mm. In all patients under consideration residual PET activity was found around the resection cavity. This was partly due to tumor invading non-resectable, so-called eloquent areas (speech, locomotion etc.), but also mirrored the diffuse nature of the disease. A fractionated dose of 1.8 Gy (5×/week) was chosen for re-irradiation [21]. The radiation dose was limited to 39.6 Gy, and prescribed to the 95% isodose according to the ICRU recommendations and following the recommendations of the ESTRO-ACROP glioblastoma guidelines to keep the total dose (primary setting 60 Gy + re-RT) below the commonly accepted 100 Gy [22–24].

### Immunohistochemistry

Nanoparticles were illustrated by Prussian blue staining for iron content. Tissue was deparaffinized and hydrated with distilled water, immersed in aqueous potassium ferrocyanide (Iron Reagent A) for 7 min, and in 25% aqueous hydrochloric acid (Iron Reagent B) for 7 min. Tissue was then rinsed with distilled water and immersed in nuclear fast red-aluminum sulfate solution 0.1% (Waldeck, Münster, Germany) for 7 min, then re-rinsed. Immunohistochemistry was performed using the avidin–biotin peroxidase technique, and staining was conducted automatically using the Dako REAL™ Detection System (K5001). Anti-CD3 (1:25, Dako, Glostrup, Denmark), anti-CD8 (1:100, Dako), anti-CD68 (1:5,000, in-house production), anti-myeloperoxidase (MPO, R&D Systems, Abingdon, UK), anti-HSP70 (1:350, Santa Cruz Biotechnology, Inc., Dallas, Texas, USA), and anti-caspase3 (1:200, Cell Signaling, Leiden, Netherlands) were used. Secondary antibodies were biotinylated goat anti-mouse and anti-rabbit immunoglobulins. Diaminobenzidine (Leica Biosystems, Nussloch, Germany) served as chromogen. The slides were counterstained using hematoxylin. Tonsil tissue was used as positive control.

### Preparation of fresh tumor cell suspensions

Fresh tumor material was obtained by ultrasonic aspiration with a CUSA EXcel® (Integra Radionics Inc., Burlington, MA, USA). Ultrasonic aspirates were collected in a sterile suction trap during tumor resection [25]. Tumor fragments were extensively washed to discard blood and suction fluid. Tumor cell suspensions were isolated as previously described [26, 27] and immediately used for further analysis.

### Multiparameter flow cytometry

All samples were stained with a panel of fluorochrome-conjugated monoclonal antibodies (mAbs) as previously described [28], and analyzed using the Navios™ flow cytometer and Kaluza 1.5 Software (Beckman Coulter, Krefeld, Germany). To determine the frequency of different lymphocyte subpopulations, CD45^+^ leukocytes were selected in a CD45 versus forward scatter channel (FSC) plot. CD45^+^ cells were then displayed in a CD14 versus sideward scatter channel (SSC) plot to identify CD14^−^ lymphocytes. To distinguish between CD3^−^CD56^+^ NK cells and CD3^+^CD56^−^ T-cells, lymphocytes were further displayed in a CD3 versus CD56 plot. CD3^+^CD56^−^ T-cells were further split into CD4^+^ and CD8^+^ T-cells. Memory CD4^+^ and CD8^+^ T-cell subsets were defined as CD45RA^−^ cells, and CD4^+^ regulatory T-cells as CD25^high^CD127^low^ T-cells. For intracellular IFN-γ and IL-4 detection, tumor cell suspensions were stimulated with phorbol 12-myristate 13-acetate (PMA, 100 ng/ml; Sigma-Aldrich, Schnelldorf, Germany) plus ionomycin (1 mg/ml; Sigma-Aldrich) in the presence of Brefeldin A (10 mg/ml; ThermoFisher Scientific) for 4 h. Cells were then stained with T-cell markers, further processed with the Fixation/Permeabilization Kit (ThermoFisher Scientific) and labeled with a PE-conjugated IFN-γ or APC-conjugated mAbs (ThermoFisher Scientific). The proportions of IFN-γ and IL-4 producing CD4^+^ and CD8^+^ CD45RA^−^ memory T-cells were determined, and results were used to calculate Th1/Th2 ratios by dividing both numbers. To determine the frequency and phenotype of different myeloid cell subpopulations, CD45^+^ leukocytes were selected in a CD45 versus FSC plot. CD45^+^ cells were then displayed in a CD11b versus SSC plot to identify CD11b^+^ myeloid cells. CD11b^+^ myeloid cell subsets were defined by CD45- and CD14-expression CD45^++/+++^CD14^high^ tumor-associated myeloid cells (TAMCs) and CD45^+/++^CD14^low^ microglia [28, 29].

## Results

### Nanoparticle instillation and subsequent thermo- and radiotherapy

**Fig. 1:**
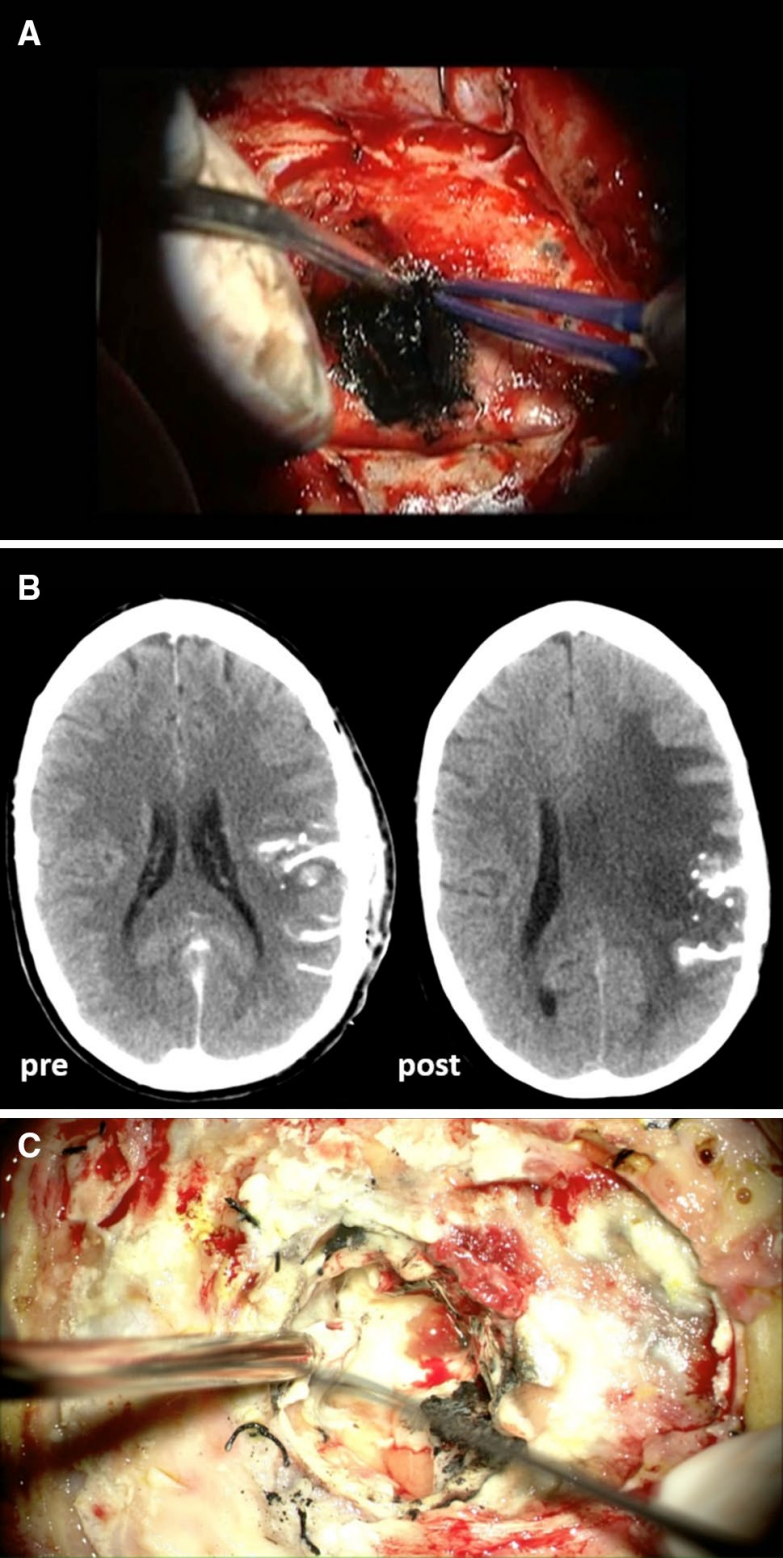
Intracavitary application of superparamagnetic iron-oxide nanoparticles. **a** Intraoperative picture of nanoparticles pasted onto the resection cavity wall “NanoPaste”. **b** Representative CT scans of a patient before (left side) and after thermotherapy (right side) with prominent edema around nanoparticle deposits. **c** Intraoperative picture of a resection cavity after removal of nanoparticles

We treated six recurrent GBM patients at a median age of 60 years with intracavitary thermotherapy using SPIONs as described in Material and Methods. Post-operative CT scan and FET-PET scan were performed and treatment parameters for the subsequent thermotherapy were simulated. The average amount of magnetic fluid instilled into the tumor cavity was 10.4 ml (range 6.7–16.3 ml) (Fig. 1a; Table 1). At the point of highest temperature, we found a mean error of 2.3 °C between simulated and measured data, which was corrected by tuning the AMF field strength. Depending on the geometry of the target area and the catheter course we chose a mean maximum temperature of 55.5 °C (± 4.1 °C) inside the treatment field at a mean magnetic field strength of 9.9 kA/m (± 2.0 kA/m). Four patients also received concurrent radiotherapy at a total dose of 39.6 Gy, fractionated with 5 × 1.8 Gy per week, whereas two patients could not receive additional radiotherapy due to maximum dose limitations or early tumor progression within 3 months after completing primary radiotherapy. When combined with irradiation, each hyperthermia session took place within 2 h of the respective radiotherapy fraction.

**Table 1:** Simulated and measured data during thermotherapy

Simulated data (n = 6)	1	2	3	4	5	6	Mean	SD
NanoTherm volume (ml)	11.6	6.7	11.3	7.6	16.3	9.1	10.4	3.5
Tumor volume (ml)	1.7	3.6	61.7	33.0	16.8	6.4	20.5	23.3
Treatment field (ml)	16.4	11.3	61.7	72.2	60.1	22.6	40.7	26.8
Temp. max. inside the treatment field (°C)	62.8	56.3	54.2	50.0	45.9	50.2	53.2	5.9
Coverage tumor volume > 39 °C (%)	100.0	99.9	83.1	53.1	99.1	54.3	81.6	22.5
H-field strength (kA/m)	11.0	10.0	12.0	9.0	6.0	11.0	9.8	2.1
**Measured data (n = 6)**								
Temp. max. inside the treatment field (°C)	59.3	56.2	60.6	54.3	53.4	49.3	55.5	4.1
H-field strenght (kA/m)	11.0	9.0	11.0	9.0	6.0	12.0	9.9	2.0

### Delayed tumor flare reactions after intracavitary thermotherapy

No major side effects were observed during active treatment. All patients exhibited sweating and reported a general sensation of warmth in the treatment area. Body temperature during thermotherapy did not exceed 38 °C with one exception. An external cooling ventilator was used to minimize body temperature elevation. However, 2–5 months later (median = 3.8 months), patients developed clinical symptoms such as headaches or worsening of pre-existing focal neurological deficits. CT scans showed a tumor flare reaction with prominent edema around the nanoparticles in all cases, and sometimes also ring-like contrast-enhancing areas, suggestive of abscess formation (Fig. 1b). All patients were treated with high dose dexamethasone and, if necessary, underwent subsequent re-surgery (n = 4) to remove the nanoparticles together with adjacent granulation tissue, which led to rapid symptom relief (Fig. 1c). Generally, patients were able to taper dexamethasone dosage after removal of nanoparticles, but still needed low dose dexamethasone throughout the follow-up period for edema control.

### Sustained inflammatory reactions after intracavitary thermotherapy

**Fig. 2:**
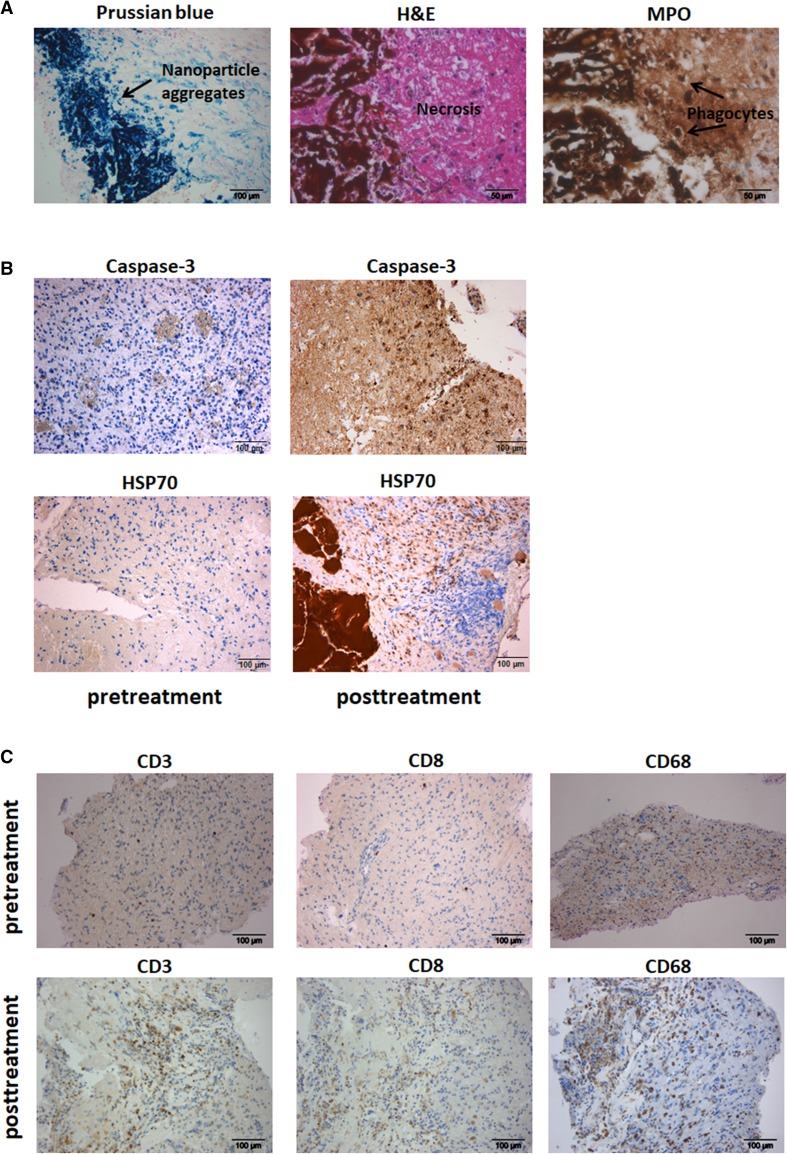
Iron staining and immunohistochemistry of histopathological sections. Sections were stained with Prussian blue, H&E and antibodies directed against myeloperoxidase (MPO), Caspase-3 and HSP70. **a** Representative pictures from tissue obtained when excessive edema led to surgical removal of nanoparticle instillation (median interval between last thermotherapy session and tissue removal = 3.8 months). Nanoparticle deposits are blue in Prussian blue and brown in H&E. Most of the nanoparticles were aggregated and located in areas of tumor necrosis. At the borders of the aggregates the nanoparticles were phagocytosed mainly by MPO-positive cells (magnification × 200 and × 400). **b** Representative pictures from a patient showing increased Caspase-3 and HSP70 expression after treatment (magnification × 200). **c** Representative immunohistochemical staining of paraffin-embedded tissue sections using mAbs against CD3, CD8 and CD68. Tumor samples showed a significant infiltration of CD3^+^, CD8^+^ and CD68^+^ cells after intracavitary thermotherapy. Pre-therapeutic tumor samples did not reveal T-cell immune cell infiltrates (magnification × 200)

**Fig. 3:**
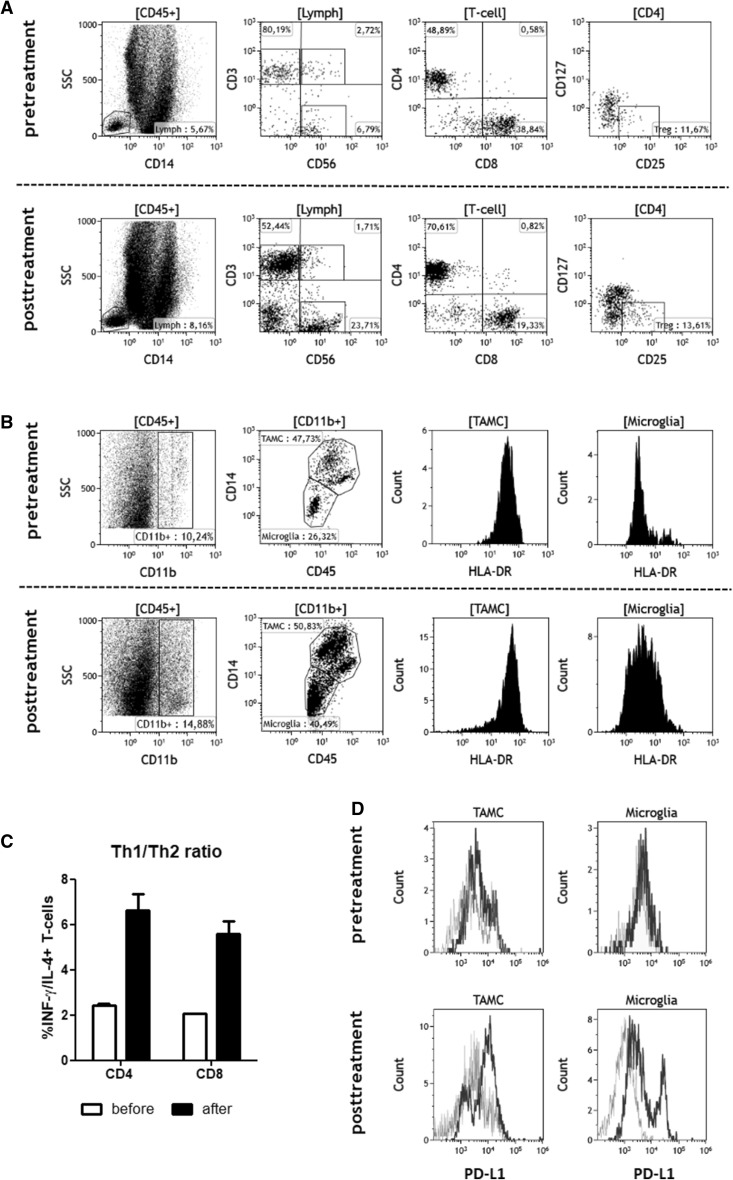
Multiparameter flow cytometry. Freshly isolated peripheral blood mononuclear cells (PBMCs) and tumor cell suspensions prepared from resected tumor material were stained using a panel of directly labelled mAbs to detect different T-cell and myeloid cell subpopulations. Gating strategies are described in detail in “Material and Methods”. **a** Pre- and posttreatment analysis of lymphocyte subpopulations, mainly CD3^+^, CD4^+^ and CD8^+^ T-cells, CD3^−^CD56^+^ NK cells and CD4^+^CD25^high^CD127^low^ regulatory T-cells. **b** Pre- and posttreatment analysis of CD45^+^CD11b^+^ myeloid cell subsets including HLA-DR expression on tumor-associated CD45^++/+++^CD14^high^ myeloid cells (TAMC) and CD45^+/++^CD14^low^ microglia. **c** Tumor cell suspensions were stimulated with PMA/Ionomycin, and CD4^+^ and CD8^+^ CD45RA^−^ memory T cells were analysed for the expression of INF-γ and IL-4 by intracellular FACS staining. Th1/Th2 ratios were calculated by dividing the proportion of IFN-γ positive T-cells by the proportion of IL-4 positive cells. **d** Pre- and posttreatment expression of PD-L1 on TAMC and microglia (black line = PD-L1; grey line = isotype control)

Histopathology of the secondarily resected tissue samples revealed large amounts of nanoparticles which were aggregated and located in necrotic areas. At the borders of the aggregates, we found a prominent infiltration of MPO-positive phagocytes with ingested nanoparticles. The surrounding tissue showed a strong upregulation of HSP70 and Caspase-3. There were no signs of tumor activity or bacterial infection in any of the analysed cases (Fig. 2a, b). In addition, we observed a significant increase of CD3^+^, CD8^+^ T-cells and CD68^+^ macrophages after thermotherapy (Fig. 2c). For a single patient, we were also able to analyse cell suspensions freshly prepared from material resected before and after radiothermotherapy for the presence of T-cell and myeloid cell subpopulations. Consistent with immunohistochemistry, we observed an increase in the frequency of lymphocytes with higher numbers of CD4^+^ T-cells and CD3^−^CD56^+^ NK-cells after treatment. The frequency of CD25^+^CD127^low^ regulatory T-cells (T_reg_) was comparable. Moreover, we detected a prominent infiltration of CD45^+^CD11b^+^ myeloid cell subsets with increased frequencies of TAMCs and microglia which showed an upregulation of the activation marker HLA-DR (MHC class II molecule) (Fig. 3a, b). Ex-vivo stimulation of tumor-infiltrating CD4^+^ and CD8^+^ memory T-cells (PMA/Ionomycin) revealed a prominent shift from a Th2 towards a Th1 phenotype with an increased intracellular ratio of IFN-γ to IL-4. Furthermore, we detected a strong upregulation of PD-L1 on TAMCs and microglia after thermotherapy (Fig. 3c, d).

### Long-term stabilisation after intracavitary thermotherapy

Patients were followed at least 3-monthly or at an individual basis with shorter time intervals according to clinical needs by clinical examinations and PET-CT or MRI scans, if nanoparticles were secondarily removed. Mean follow-up time was 11.8 ± 9.3 months. Tumor progression was defined according to RANO criteria. Four patients were re-operated to remove nanoparticles. Patients did not receive salvage chemotherapy or other antitumor treatment after intracavitary thermotherapy and radiotherapy. As shown in Table 2, we observed two patients with durable treatment responses > 23 months with one patient who was still alive with an overall survival greater than 29 months after the last patient visit (14-May-2018). Median PFS (mPFS) was 6.25 months and median OS (mOS) was 8.15 months for all patients. When considering only patients treated at first recurrence, mOS was 23.9 months, whereas patients treated at second recurrence or later had a mOS of 7.1 months.

**Table 2:** Patients characteristics and follow-up data

Patient	Age, sex	Recurrence	MGMT methylation	Treatment	NP removal	PFS (months)	OS (months)	Status
1	42, f	1	1	NT + RX	1	29.1	29.1	0
2	59, m	1	0	NT + RX	1	10.6	23.9	1
3	75, f	1	0	NT	1	5.3	7.1	1
4	60, m	2	0	NT + RX	0	6.9	7.1	1
5	65, m	2	1	NT + RX	1	5.6	9.2	1
6	42, m	4	0	NT	0	2.8	3.7	1

*NP* nanoparticle, *NT* nanotherapy, *RT* radiotherapy, *0* alive, *1* dead

Last update 14 May 2018

## Discussion

Currently, there are few treatment options in recurrent glioblastoma, and novel approaches are needed.

If patients are in good clinical condition and recurrences are circumscribed, repeat surgery is often recommended followed by a second course of radiotherapy and chemotherapy rechallenge [30]. Hyperthermia with or without concomitant radiotherapy has likewise been suggested as a possible option. One randomized study using helical microwave antennas and interstitial brachytherapy demonstrated improved survival in the treatment group, but also suffered from several technical problems [31].

“NanoPaste” has the potential of overcoming these earlier challenges. Coating the inner walls of a glioma resection cavity with SPIONs can easily be performed and has several advantages over classical stereotactic instillation. First, we were able to homogeneously distribute the nanoparticles within the target area under direct visual control. Second, there was no need to inject magnetic nanoparticles at multiple sites or through multiple trajectories, thereby circumventing the problem of leakage or reflow from the needle tracts. Third, treatment parameters could be easily simulated after mapping nanoparticle density and distribution in a CT scan, and temperatures measured during therapy were simply adaptable by adjusting the magnetic field strength.

Therapy simulation is still facing a number of basic problems mainly originating from the lack of three-dimensional measuring techniques for tissue perfusion and temperature development under the application of an AMF. Current developments will need several years (J Sachs, Technical University Ilmenau, Germany; personal communication). Considering our still rudimentary possibilities in this field, we observed a remarkably good correlation between simulated and measured temperatures during treatment with deviations of around 2 °C.

There were no major side effects during active treatment, but with a delay of 2–5 months, perifocal edema significantly increased around the nanoparticle deposits leading to a clinical deterioration of all patients. Probably because all patients were treated within a limited range of peak temperatures inside the treatment field (± 4.1 °C), cerebral edema developed within similar time frames (median 3.8 months). There was no correlation between the volume of nanoparticles and the extent of cerebral edema. Cerebral edema could be temporarily controlled by dexamethasone treatment, but two-thirds of the patients additionally needed neurosurgical interventions to remove nanoparticle deposits together with adjacent granulation tissue. After that, patients were able to slowly taper dexamethasone doses.

As cerebral edema occurred early both in patients with and without radiotherapy, a significant role of hyperthermia in edema development is to be assumed, although radiotherapy applied within a short time interval may still add to this process [32]. Neurotoxicity directly attributed to re-irradiation is difficult to determine and has been found to be dose-dependent, but radiation—induced necrosis after normofractionated radiotherapy seems to be a rare event, and to develop as a late complication [33–36].

Histopathology delivered several explanations for the generation of cerebral edema. We found massive tumor necrosis in the areas next to large nanoparticle aggregates. At the borders of the necrotic zone, apoptotic cell death, identified by Caspase-3 activation, and enhanced expression of HSP70 could be detected. These observations correlate closely with the temperature gradient induced by our approach: Temperature decreases from the inner to the outer zone of the treatment field with core temperatures above 45 °C known to induce necrotic cell death and rim temperatures between 42 and 45 °C which usually cause apoptotic cell death. Temperatures above 40 °C are necessary to stimulate the expression of HSPs which are known to be upregulated to protect cells against apoptosis [37, 38].

Just as tumor necrosis plays a pivotal role in the attraction and activation of leucocytes, we found an increased infiltration of phagocytes with subsequent phagocytosis of necrotic debris and nanoparticles. Phagocytic cells also expressed myeloperoxidase, a well-known enzyme with strong pro-oxidative and proinflammatory properties which is mainly released by activated polymorphonuclear cells [39]. In addition, we could observe enhanced infiltration by T-cells, NK-cells, TAMCs, and microglia. Profiling of these cells revealed a switch from an immunosuppressive Th2 to a pro-inflammatory Th1 phenotype with increased ratios of INF-y to IL-4 in tumor-infiltrating CD4^+^ and CD8^+^ memory T-cells. TAMCs and microglia showed an upregulation of activation markers such as MHC class II molecules (HLA-DR) and PD-L1.

As glioblastomas are characterized by a strong immunosuppressive microenvironment that effectively resists immune attacks, our data indicate that combined intracavitary thermotherapy and radiotherapy could be an efficient tool to convert a non-inflamed tumor into an inflamed tumor [40]. Recent work has clearly demonstrated that tumors with an inflamed phenotype like prominent T-cell infiltrates, a type I interferon signature and enhanced PD-L1 expression might be more susceptible towards checkpoint inhibition or other immunotherapeutic approaches than non-inflamed tumors [41].

Accordingly, HSPs such as HSP70 which are upregulated during hyperthermia serve as key players in linking innate and adaptive immunity through the upregulation of MHC class I molecules on tumor cells, activation of pro-inflammatory cytokines and adhesion molecules facilitating immune cell trafficking into the tumor tissue, activation of NK cells, and enhanced presentation of antigenic HSP-peptide complexes to CD4^+^ and CD8^+^ T-cells by dendritic cells [42, 43].

When considering immunotherapy approaches in combination with local hyperthermia and radiotherapy, checkpoint inhibitors such as anti-PD-L1 mAbs or adoptive transfer therapies using HSP70-specific NK-cells might be promising candidates [44, 45].

Even though the sample size of our case series is far too small to draw any reliable conclusions, earlier interventions seem to be associated with more favourable outcomes. This is supported by the fact that glioblastoma patients who were treated with “NanoPaste” at first recurrence had longer survival times than those treated at second recurrence or even later (mOS = 23.9 months vs. 7.1 months). Moreover, we observed durable treatment responses with prolonged overall survival predominantly in patients who received both treatment modalities. A synergistic treatment effect has been illustrated in several other preclinical and clinical studies demonstrating that the combination of local hyperthermia and radiation is significantly more effective in tumor growth reduction than radiation alone [46–48].

Glioblastoma patients with borderline intracranial compliance are endangered by additional brain edema. However, as edema formation may also indicate immunological effects, further investigations of the treatment conditions such as the target temperature and heating period during intracavitary thermotherapy are needed to determine the most suitable conditions for the induction of antitumor immune responses either without inducing persistent perifocal edema, or including strategies for edema control without abrogating immunological effects. This will be of utmost importance, as the currently used high doses of corticosteroids strongly interfere with the efficacy of immunotherapeutic approaches, and patients are at risk of developing typical side effects of longer term steroid intake. At the current state, hyperthermia should be limited to patients not in need of corticosteroids before treatment starts.

To further proceed with the “NanoPaste” technique, we are currently designing a phase-I study enrolling patients at first recurrence of glioblastoma with a Karnofsky performance status > 70 who are eligible for re-surgery and are not dependent on corticosteroids. Patients will be allocated to 3 thermal dose cohorts of 45 °C, 50 °C and 55 °C, respectively. Primary outcome measures will be safety and tolerability. Secondary outcome measures will be clinical antitumor activity as well as biomarker assessment at serial time points and its correlation to the clinical response.

## Conclusion

Intracavitary thermotherapy with superparamagnetic iron oxide nanoparticles combined with radiotherapy could be a promising treatment modality in glioblastoma patients at first recurrence, and our preliminary results warrant further investigations in a prospective phase-I study.
